# Supplementary material for: Maternal gut and vaginal microbiota, alcohol use during pregnancy, and infant fetal alcohol spectrum disorder diagnosis in a South African cohort
Source: medRxiv. 2026 Aug 6:2026.08.04.26359606. Preprint. [Version 1] doi: 10.64898/2026.08.04.26359606 (PMC13484794; doi:10.64898/2026.08.04.26359606)
Supplement: Supplementary file 1 [file media-1.pdf]

Maternal gut and vaginal microbiota, alcohol use during pregnancy, and infant fetal alcohol spectrum disorder diagnosis in a South African cohort

Supporting Tables, Data & Figures

Lauren C. Martin[1,2,*], Natasha Kitchin[1,2], Jacqueline S. Womersley[1,2,3,4], Kristien Nel Van Zyl[5], Anna-Susan Marais[1], Marlene M. de Vries[1], Matthew J. Dalby[6,7], Raymond Kiu[6,7,8], Lindsay J. Hall[6,7,8,9], Philip A. May[10], Soraya Seedat[1,2], Sian M.J. Hemmings[1,2,*]

[1] Department of Psychiatry, Faculty of Medicine and Health Sciences, Stellenbosch University, Cape Town, South Africa

[2] South African Medical Research Council/Stellenbosch University Genomics of Brain Disorders Research Unit, Stellenbosch University, Cape Town, South Africa

[3] Neuroscience Institute, University of Cape Town, Cape Town, South Africa

[4] Department of Human Biology, Faculty of Health Sciences, University of Cape Town, Cape Town, South Africa

[5] African Microbiome Institute, Division of Molecular Biology and Human Genetics, Department of Biomedical Sciences, Faculty of Medicine and Health Sciences, Stellenbosch University, Cape Town, South Africa

[6] Institute of Microbiology and Infection, University of Birmingham, Birmingham, United Kingdom

[7] Department of Microbes, Infection and Microbiomes, School of Infection, Inflammation and Immunology, College of Medicine and Health, University of Birmingham, Birmingham, United Kingdom

[8] Food, Microbiome and Health, Quadram Institute Bioscience, Norwich Research Park, Norwich, United Kingdom

[9] Norwich Medical School, University of East Anglia, Norwich Research Park, Norwich, United Kingdom

[10] Department of Nutrition, Gillings School of Global Public Health, Nutrition Research Institute, University of North Carolina, United States of America

Martin (lcmartin@sun.ac.za); Kitchin (natashak@sun.ac.za); Womersley (jsw1@sun.ac.za); Nel Van Zyl (knvz@sun.ac.za); Marais (asmarais@sun.ac.za); de Vries (mmdevries@sun.ac.za); Dalby (m.j.dalby@bham.ac.uk); Kiu (Raymond.Kiu@quadram.ac.uk); Hall (l.hall.3@bham.ac.uk); May (pmay@unm.edu); Seedat (sseedat@sun.ac.za); Hemmings (smjh@sun.ac.za)

Corresponding authors: Lauren C. Martin & Sian M.J. Hemmings*

Correspondence address: Department of Psychiatry, Faculty of Medicine and Health Sciences, Stellenbosch University, PO Box 241, Cape Town 8000, South Africa

Tel: +27 21 938 9321; Email: lcmartin@sun.ac.za; smjh@sun.ac.za

Table S1: Covariate adjustment sets for maternal fecal and vaginal microbiota alcohol use and infant FASD diagnosis analyses.

Diversity metric	Covariate	Effect size	*p*-value
**Alcohol use maternal fecal microbiota analysis (n = 207)**			
Bray-Curtis dissimilarity	GA	0.0164	0.001
Bray-Curtis dissimilarity	BSS	0.0210	0.003
Bray-Curtis dissimilarity	Educational attainment	0.0170	0.008
Bray-Curtis dissimilarity	Extraction batch	0.0673	0.023
Observed richness	BSS	3.4500	0.034
Bray-Curtis dissimilarity	BMI	0.0107	0.042
Observed richness	Smoking	3.9000	0.050
~ **AUDIT/HAU** + GA + BSS + Educational attainment + Extraction batch + BMI + Smoking			
**Infant FASD diagnosis maternal fecal microbiota analysis (n = 168)**			
Bray-Curtis dissimilarity	GA	0.0183	0.007
Bray-Curtis dissimilarity	Educational attainment	0.0166	0.012
Bray-Curtis dissimilarity	Extraction batch	0.0854	0.014
Bray-Curtis dissimilarity	BSS	0.0257	0.020
Bray-Curtis dissimilarity	Antibiotics	0.0131	0.048
Shannon diversity	BSS	3.5100	0.032
Observed richness	HIV	4.3600	0.038
Observed richness	BSS	3.4700	0.033
Bray-Curtis dissimilarity	BMI	0.0139	0.040
**~ Infant diagnosis group** + GA + Educational attainment + Extraction batch + Antibiotics + BSS + HIV + BMI			
**Alcohol use maternal vaginal microbiota analysis (n = 28)**			
Observed richness	Age	1.5000	0.009
**~ AUDIT/HAU + Age**			
**Infant FASD diagnosis maternal vaginal microbiota analysis (n = 15)**			
Observed richness	Smoking	9.6100	0.008
Observed richness	Age*	-1.1590	0.050
**~ Infant diagnosis group + Smoking**			

*Variable not included due to constrained sample size and the risk of statistical overfitting.
AUDIT – Alcohol Use Disorders Identification Test; BMI – body mass index; BSS – Bristol Stool Scale; GA – Gestational age; HAU – hazardous alcohol use; HIV - Human Immunodeficiency Virus

Table S2: Characteristics of participants in the cross-sectional maternal fecal and vaginal microbiota infant FASD diagnosis investigation.

	Participants that provided fecal samples					Participants that provided vaginal swab samples				
	All participants (n = 168)	FASD[a] (n = 80)	Not FASD (n = 88)	t/W/X^2 value	*p*-value	All participants (n = 15)	FASD[a] (n = 10)	Not FASD (n = 5)	t/W/X^2 value	*p*-value
**Clinical and socio-demographic information**										
**Age (years)**	26.00 (22.00 – 27.21)	25.50 (22.00 – 33.00)	26.00 (22.00 – 31.00)	3651	0.678	29.47 (5.33)	30.10 (5.36)	28.20 (5.63)	0.63	0.549
**BMI (kg/m^2)**	24.90 (21.07 – 30.98)	23.65 (21.00 – 28.95)	25.85 (21.20 – 31.78)	3085	0.168	22.67 (2.98)	22.30 (3.19)	23.41 (2.68)	-0.71	0.496
**Gestational age at sample collection (weeks)**	19.00 (14.00 – 26.00)	20.00 (14.00 – 26.25)	18.00 (13.50 – 24.00)	3873.50	0.262	38.13 (1.41)	38.30 (1.25)	37.80 (1.79)	0.56	0.596
**Antibiotic use in past six months, *n (%)***	25 (14.88)	10 (12.50)	15 (17.05)	0.37	0.542	2 (13.33)	2 (20.00)	0.00	-	0.524
**HIV-positive serostatus, *n (%)***	6 (3.57)	3 (3.75)	3 (3.41)	1.07e-29	1.000	2 (13.33)	2 (20.00)	0.00	-	0.524
**Maternal education (highest grade completed)**	9.00 (7.00 – 11.00)	8.00 (7.00 – 10.00)	9.00 (8.00 – 11.00)	2700.50	**0.009**	8.60 (2.26)	8.10 (2.51)	9.60 (1.34)	-1.51	0.156
**Employment status, *n (% employed)***	67 (39.88)	30 (37.50)	37 (42.05)	0.20	0.658	5 (33.33)	4 (40.00)	1 (20.00)	-	0.600
**Alcohol and substance use during index pregnancy**										
**AUDIT score**	11.00 (0.00 – 17.00)	15.00 (8.00 – 21.00)	4.50 (0.00 – 12.25)	5074.50	**< 0.001**	17.27 (10.68)	20.80 (10.49)	10.20 (7.63)	-2.23	**0.048**
**HAU, *n (%)*[b]**	108 (64.29)	68 (85.00)	40 (45.45)	26.85	**< 0.001**	13 (86.67)	10 (100.00)	3 (60.00)	-	0.600
**PEth level (ng/mL)[c,d]**	15.00 (0.00 – 75.33)	44.84 (5.75 – 210.50)	27.11 (5.00 – 23.84)	3955	**< 0.001**	107.00 (79.00 – 153.50)	107.00 (79.00 – 153.50)	-	-	-
**EtG level (pg/mg)[e,f]**	0.00 (0.00 – 30.00)	19.00 (0.00 – 76.50)	0.00 (0.00 – 6.00)	3467	**< 0.001**	0.00 (0.00 – 36.25)	0.00 (0.00 – 36.25)	-	-	-
**Smoked during index pregnancy, *n (%)***	111 (66.07)	59 (73.75)	52 (59.09)	3.39	0.066	12 (80.00)	8 (80.00)	4 (80.00)	-	1.000
**Characteristics of child born from index pregnancy**										
**Sex, *n (% male)***	86 (51.19)	45 (56.25)	41 (46.59)	0.00	1.000	7 (46.67)	4 (40.00)	3 (30.00)	-	0.608

Continuous data are summarised as mean (standard deviation) if normally distributed, and as median (interquartile range) if non-normally distributed, unless otherwise specified. Categorical data are summarised as counts and percentages.

[a] Participants were included in the FASD group on the basis of their infant receiving a confirmed diagnosis on the FASD spectrum.

[b] Participants were considered to have consumed alcohol hazardously during pregnancy if AUDIT score ≥ 7.

[c] PEth levels were available for 72 participants in the FASD group and 79 participants in the Not FASD group for the fecal analysis.

[d] PEth levels were available for 5 participants in the FASD group and 0 participants in the Not FASD group for the vaginal analysis.

[e] EtG levels were available for 69 participants in the FASD group and 71 participants in the Not FASD group for the fecal analysis.

[f] EtG levels were available for 5 participants in the FASD group 0 participants in the Not FASD group for the vaginal analysis.

AUDIT - Alcohol Use Disorders Identification Test; BMI - body mass index; EtG – Ethyl Glucuronide; FASD – fetal alcohol spectrum disorders; HAU – hazardous alcohol consumption; HIV - Human Immunodeficiency Virus; PEth – Phosphatidylethanol

Table S3: Phylum-level median relative bacterial abundance of maternal fecal samples in alcohol use (n = 207) and infant FASD diagnosis analysis (n = 168).

**Alcohol use fecal analysis (n = 207)**		**FASD diagnosis fecal analysis (n = 168)**	
**Phylum**	**Median relative abundance (%)**	**Phylum**	**Median relative abundance (%)**
Bacillota	45.10	Bacillota	45.40
Bacteroidota	38.50	Bacteroidota	39.00
Pseudomonadota	6.96	Pseudomonadota	6.49
Actinomycetota	1.65	Actinomycetota	1.62
Thermodesulfobacteriota	0.57	Thermodesulfobacteriota	0.54
Verrucomicrobiota	0.01	Verrucomicrobiota	0.009
Cyanobacteriota	0.008	Cyanobacteriota	0.007

Table S4: Median relative bacterial abundance of top ten genera in maternal fecal samples in alcohol use (n = 207) and infant FASD diagnosis analysis (n = 168).

**Alcohol use fecal analysis (n = 207)**		**FASD diagnosis fecal analysis (n = 168)**	
**Genus**	**Median relative abundance (%)**	**Genus**	**Median relative abundance (%)**
Segatella	23.30	*Segatella*	21.30
Faecalibacterium	4.42	*Faecalibacterium*	4.55
Succinivibrio	3.86	*Oscillospiraceae UCG-002*	3.70
Oscillospiraceae UCG-002	3.61	*Succinivibrio*	3.56
Bacteroides	3.09	*Bacteroides*	3.32
Dialister	1.90	*Dialister*	1.57
Leyella	1.51	*Leyella*	1.21
Lachnospiraceae NK4A136 group	1.16	*Lachnospiraceae NK4A136* group	1.23
Roseburia	1.08	*Roseburia*	1.07
Sutterella	1.06	*Oscillospiraceae UCG-005*	1.06

The most abundant phyla across all the fecal samples included in the FASD diagnosis analysis (n = 168) were *Bacillota* (formerly *Firmicutes*), *Bacteroidota* (formerly *Bacteroidetes*), *Pseudomonadota*, *Actinomycetota*, *Thermodesulfobacteriota*, *Verrucomicrobiota* and *Cyanobacteriota* (**Table S2**).

At the genus-level, the fecal microbiota of the 168 participants who provided fecal samples and had an infant who had undergone diagnostic assessment for FASD (Aim 2) was dominated by *Segatella* species, which was present at a median relative abundance of 21.3% (**Table S3**). In addition, and listed in order of decreasing median abundance, *Faecalibacterium* (4.6%), *Oscillospiraceae UCG-002* (3.7%), *Succinivibrio* (3.6%), *Bacteroides* (3.3%), *Dialister* (1.6%), *Lachnospiraceae NK4A136* group (1.2%), *Leyella* (1.2%), *Roseburia* (1.1%) and *Oscillospiraceae UCG-005* (1.1%), were among the remaining top ten most abundant bacterial genera present.

Table S5: Maternal fecal genera with the strongest nominal joint associations (*p*-joint) with self-reported alcohol use measures (AUDIT score; AUDIT ≥ 7) and infant FASD diagnostic group, identified using MaAsLin3's linear mixed-effects abundance model and logistic prevalence model.

	Abundance model							Prevalence model						
**Genus**	**Coef[a]**	**SE**	***p*-abun**	***q*-abun**	**N[*]**	***p*-joint**	***q*-joint**	**Coef[b]**	**SE**	***p*-abun**	***q*-abun**	**N[*]**	***p*-joint**	***q*-joint**
**Continuous AUDIT score (n = 207)**														
Victivallis	-0.408	0.172	**0.017**	0.402	111	0.002	0.319	-0.566	0.168	**0.001**	0.306	111	0.002	0.319
Butyricimonas	0.011	0.160	0.986	0.999	135	0.004	0.319	-0.535	0.172	**0.002**	0.306	135	0.004	0.319
Erysipelatoclostridiaceae UCG-004	0.096	0.211	0.696	0.946	115	0.006	0.319	-0.515	0.174	**0.003**	0.306	115	0.006	0.319
Erysipelotrichaceae UCG-003	0.196	0.299	0.542	0.918	77	0.007	0.319	0.490	0.168	**0.004**	0.306	77	0.007	0.319
Oscillospiraceae NK4A214 group	-0.339	0.119	**0.004**	0.313	198	0.009	0.326	-0.080	0.360	0.825	0.976	198	0.009	0.326
Oscillospiraceae UCG-005	-0.466	0.166	**0.005**	0.313	199	0.010	0.326	-0.184	0.379	0.627	0.943	199	0.010	0.326
Oscillospiraceae UCG-002	-0.388	0.142	**0.006**	0.334	199	0.012	0.341	-0.058	0.409	0.887	0.986	199	0.012	0.341
Butyricicoccaceae UCG-009	-0.165	0.203	0.382	0.832	130	0.017	0.341	-0.454	0.173	0.009	0.334	130	0.017	0.341
Lachnospiraceae [Eubacterium] oxidoreducens group	0.492	0.176	**0.010**	0.334	66	0.019	0.341	-0.371	0.174	**0.033**	0.450	66	0.019	0.341
Mediterraneibacter	0.368	0.135	**0.011**	0.334	183	0.022	0.341	-0.340	0.258	0.188	0.686	183	0.022	0.341
Intestinibacter	-0.432	0.181	**0.016**	0.402	103	0.033	0.408	0.036	0.161	0.823	0.976	103	0.033	0.408
Bacteroides	0.231	0.232	0.351	0.813	202	0.035	0.411	-1.933	0.816	**0.018**	0.402	202	0.035	0.411
Lachnospiraceae [Eubacterium] xylanophilum group	0.029	0.208	0.942	0.991	109	0.040	0.411	-0.380	0.164	**0.020**	0.402	109	0.040	0.411
Ruminococcus	0.040	0.131	0.841	0.979	199	0.047	0.426	-1.212	0.536	**0.024**	0.421	199	0.047	0.426
Desulfovibrio	-0.171	0.124	0.144	0.649	171	0.048	0.430	-0.473	0.210	**0.025**	0.421	171	0.048	0.430
**HAU[c] (n = 207)**														
Victivallis	-0.540	0.341	0.098	0.641	111	0.008	0.359	-1.016	0.354	**0.004**	0.346	111	0.008	0.359
Erysipelotrichaceae UCG-003	0.173	0.660	0.834	0.987	77	0.014	0.359	1.063	0.393	**0.007**	0.346	77	0.014	0.359
Lachnospiraceae [Eubacterium] oxidoreducens group	0.962	0.370	**0.016**	0.430	66	0.032	0.420	-0.394	0.357	0.269	0.765	66	0.032	0.420
Intestinibacter	-0.918	0.388	**0.017**	0.430	103	0.034	0.420	0.323	0.345	0.350	0.821	103	0.034	0.420
Paraprevotella	0.827	0.328	**0.019**	0.430	99	0.038	0.420	-0.397	0.346	0.251	0.765	99	0.038	0.420
Roseburia	0.635	0.253	**0.021**	0.443	197	0.042	0.435	-1.018	0.865	0.239	0.753	197	0.042	0.435
Clostridium	0.147	0.353	0.751	0.977	179	0.043	0.435	-1.415	0.617	0.022	0.443	179	0.043	0.435
**Infant FASD diagnostic category (n = 168)**														
Lachnospiraceae CAG-56	-0.415	0.362	0.423	0.872	73	0.009	0.458	-0.962	0.339	**0.005**	0.438	73	0.009	0.458
Lachnospiraceae [Eubacterium] xylano philum group	-0.078	0.383	0.905	0.994	94	0.009	0.458	-0.957	0.339	**0.005**	0.438	94	0.009	0.458
Asteroleplasma[#]	-	-	-	-	48	0.010	0.458	-0.956	0.372	**0.010**	0.438	48	0.010	0.458
Bifidobacterium	-0.913	0.367	**0.035**	0.558	143	0.010	0.458	-1.540	0.550	**0.005**	0.438	143	0.010	0.458
Lachnospiraceae ND3007 group	-0.741	0.255	**0.019**	0.508	140	0.014	0.458	-1.242	0.459	**0.007**	0.438	140	0.014	0.458
Butyribacter	-0.627	0.455	0.274	0.802	79	0.017	0.458	-0.952	0.362	**0.008**	0.438	79	0.017	0.458
Negativibacillus	0.794	0.340	**0.009**	0.438	85	0.018	0.458	-0.557	0.321	0.083	0.623	85	0.018	0.458

Ruminococcaceae [Eubacterium] siraeum group	0.186	0.378	0.414	0.868	123	0.019	0.458	-1.042	0.403	**0.010**	0.438	123	0.019	0.458
Coriobacteriaceae UCG-003[#]	-	-	-	-	26	0.021	0.458	-1.161	0.501	**0.021**	0.508	26	0.021	0.458
Fusicatenibacter	-0.806	0.271	**0.015**	0.496	138	0.029	0.519	-0.631	0.429	0.142	0.682	138	0.029	0.519
Catenibacterium	-0.804	0.278	**0.018**	0.508	124	0.036	0.551	0.224	0.374	0.549	0.932	124	0.036	0.551
Christensenellaceae R-7 group	-0.060	0.401	0.873	0.994	157	0.042	0.551	-2.698	1.173	**0.021**	0.508	157	0.042	0.551

Co-efficients (Coef) represent the difference in each group relative to the Control group for the categorical measures.
[a] Abundance co-efficient representing the log2 fold per one-unit change in continuous measure or between-group difference for a given feature, if present.
[b] Prevalence co-efficient representing the log-odds ratio of a feature's presence between groups or for every one-unit increase in continuous measure.
[c] Participants were considered to have consumed alcohol hazardously during pregnancy if AUDIT score ≥ 7.
* Number of samples that did not have a zero value.
[#] MaAsLin3 linear abundance model could not be fitted.
AUDIT – Alcohol Use Disorders Identification Test; FASD – fetal alcohol spectrum disorder; HAU – hazardous alcohol use; SE - standard error; q-value – Benjamini-Hochberg false discovery rate (FDR)-adjusted p-value

Table S6: *PICRUSt2*-predicted maternal fecal microbiota KEGG pathways nominally associated with alcohol use and infant FASD diagnosis

Outcome	KEGG pathway	Category	Sub-category	Pathway description	Coef (β)*	SE	*p*-value	*q*-value
**AUDIT score (n = 207)**	ko00552	Metabolism	Glycan biosynthesis and metabolism	Teichoic acid biosynthesis	-0.030	0.009	0.007	0.363
	ko00040	Metabolism	Carbohydrate metabolism	Pentose and glucuronate interconversions	0.033	0.015	0.017	0.479
	ko99987	-	-	-	0.102	0.046	0.026	0.532
	ko00785	Metabolism	Metabolism of cofactors and vitamins	Lipoic acid metabolism	-0.133	0.058	0.027	0.532
	ko00591	Metabolism	Lipid metabolism	Linoleic acid metabolism	-0.248	0.112	0.031	0.532
	ko03051	-	-	-	-0.020	0.007	0.032	0.532
	ko04614	Organismal systems	Endocrine system	Renin-angiotensin system	-0.173	0.079	0.033	0.532
	ko03010	Genetic information processing	Translation	Ribosome	-0.022	0.008	0.033	0.532
	ko03011	-	-	-	-0.022	0.008	0.033	0.532
	ko99977	-	-	-	0.023	0.011	0.033	0.532
	ko00565	Metabolism	Lipid metabolism	Ether lipid metabolism	-0.230	0.106	0.033	0.532
	ko00410	Metabolism	Metabolism of other amino acids	beta-Alanine metabolism	0.021	0.010	0.034	0.533
	ko00970	Genetic information processing	Translation	Aminoacyl-tRNA biosynthesis	-0.023	0.009	0.036	0.556
	ko99994	-	-	-	0.0333	0.017	0.037	0.561
	ko00670	Metabolism	Metabolism of cofactors and vitamins	One carbon pool by folate	-0.023	0.009	0.038	0.561
	ko00480	Metabolism	Metabolism of other amino acids	Glutathione metabolism	0.023	0.012	0.040	0.561
	ko03420	Genetic information processing	Replication and repair	Nucleotide excision repair	-0.021	0.008	0.041	0.561
	ko00514	Metabolism	Glycan biosynthesis and metabolism	Other types of O-glycan biosynthesis	-0.186	0.090	0.043	0.561
	ko03016	-	-	-	-0.020	0.008	0.045	0.561
	ko00052	Metabolism	Carbohydrate metabolism	Galactose metabolism	0.016	0.009	0.045	0.561
	ko03430	Genetic information processing	Replication and repair	Mismatch repair	-0.022	0.009	0.046	0.561
	ko99996	-	-	-	0.022	0.012	0.047	0.561
	ko00515	Metabolism	Glycan biosynthesis and metabolism	Mannose type O-glycan biosynthesis	-0.163	0.080	0.048	0.561
**HAU# (n = 207)**	ko04614	Organismal systems	Endocrine system	Renin-angiotensin system	-0.348	0.169	0.040	0.593
	ko00440	Metabolism	Metabolism of other amino acids	Phosphonate and phosphinate metabolism	0.090	0.044	0.048	0.593
**Infant FASD diagnostic group (n = 168)**	ko05150	Human diseases	Infectious disease: bacterial	*Staphylococcus aureus* infection	-0.418	0.135	0.003	0.259
	ko00906	Metabolism	Metabolism of terpenoids and polyketides	Carotenoid biosynthesis	-0.342	0.138	0.016	0.523
	ko00552	Metabolism	Glycan biosynthesis and metabolism	Teichoic acid biosynthesis	-0.059	0.020	0.016	0.523
	ko99985	-	-	-	0.083	0.036	0.019	0.553
	ko00997	Metabolism	Biosynthesis of other secondary metabolites	Biosynthesis of various other secondary metabolites	-0.553	0.240	0.024	0.553
	ko00542	Metabolism	Glycan biosynthesis and metabolism	O-Antigen repeat unit biosynthesis	-0.300	0.131	0.029	0.553
	ko00572	Metabolism	Glycan biosynthesis and metabolism	Arabinogalactan biosynthesis - *Mycobacterium*	-0.293	0.131	0.030	0.553
	ko00515	Metabolism	Glycan biosynthesis and metabolism	Mannose type O-glycan biosynthesis	-0.341	0.158	0.036	0.553
	ko04138	Cellular processes	Transport and catabolism	Autophagy - yeast	-0.233	0.109	0.040	0.558

Differential abundance testing performed using MaAsLin3 v.0.99.16 linear models with total sum scaling and log transformation.
Statistical significance defined as q ≤ 0.10. Results with p < 0.05 and q > 0.10 are reported as nominal associations.
* Coefficient (Coef or β) represents the difference in the log-transformed relative abundance of the reported KEGG pathway per one-unit increase in continuous measures or between groups.
# Participants were considered to have consumed alcohol hazardously during pregnancy if AUDIT score ≥ 7.
AUDIT – Alcohol Use Disorders Identification Test; FASD – fetal alcohol spectrum disorder; HAU – hazardous alcohol use; SE - standard error; q-value – Benjamini-Hochberg false discovery rate (FDR)-adjusted p-value

Table S7: Maternal fecal microbiota MetaCyc pathways nominally associated with maternal alcohol use measures and infant FASD diagnosis.

Outcome	MetaCyc ID	Class	Sub-class	Pathway	Co-eff (β)*	SE	*p*-value	*q*-value
**AUDIT score (n = 207)**	LIPASYN-PWY	Degradation, Utilization, Assimilation	Fatty acid and lipid degradation	Phospholipases	-0.349	0.114	0.003	0.121
	PWY-8187	Generation of precursor metabolites and energy; Degradation, Utilization, Assimilation	Fermentation; amino acid degradation	L-arginine degradation XIII (reductive Stickland reaction)	-0.216	0.073	0.005	0.148
	POLYAMINSYN3-PWY	Biosynthesis	Amide, Amidine, Amine, and Polyamine Biosynthesis	Superpathway of polyamine biosynthesis II	-0.178	0.063	0.008	0.179
	PWY-6471	Biosynthesis; Detoxification	Cell structure biosynthesis; Antibiotic resistance	Peptidoglycan biosynthesis IV (*Enterococcus faecium*)	-0.215	0.085	0.016	0.259
	PWY-4984	Degradation, Utilization, Assimilation	Inorganic nutrient metabolism	Urea cycle	-0.200	0.083	0.021	0.281
	PWY-6803	Biosynthesis	Fatty acid and lipid biosynthesis	Phosphatidylcholine acyl editing	-0.250	0.108	0.027	0.313
	PWY-7282	Biosynthesis	Co-factor, carrier & vitamin biosynthesis	4-amino-2-methyl-5-diphosphomethylpyrimidine biosynthesis II	0.253	0.123	0.036	0.347
	PWY-5265	Biosynthesis	Cell structure biosynthesis	Peptidoglycan biosynthesis II (*staphylococci*)	-0.218	0.100	0.036	0.347
	PWY0-845	Biosynthesis	Co-factor, carrier & vitamin biosynthesis	Superpathway of pyridoxal 5'-phosphate biosynthesis and salvage	0.265	0.134	0.044	0.367
	PWY-7663	Biosynthesis	Fatty acid and lipid biosynthesis	Gondoate biosynthesis (anaerobic)	0.014	0.009	0.045	0.367
**HAU# (n = 207)**	PWY-7820	Biosynthesis	Cell structure biosynthesis	Teichuronic acid biosynthesis (*B. subtilis* 168)	-1.594	0.453	**< 0.001**	**0.043**
	FUC-RHAMCAT-PWY	-	-	Superpathway of fucose and rhamnose degradation	0.213	0.095	0.023	0.273
**Infant FASD diagnostic group (n = 168)**	PWY-6470	Biosynthesis; Detoxification	Cell-Structure-Biosynthesis; Antibiotic-Resistance	Peptidoglycan biosynthesis V (β-lactam resistance)	-0.335	0.100	0.002	0.104
	PWY-7399	Degradation, Utilization, Assimilation	Inorganic nutrient metabolism	Methylphosphonate degradation II	-0.953	0.306	0.003	0.110
	PWY-6383	Biosynthesis	Co-factor, carrier & vitamin biosynthesis; Polyprenyl biosynthesis	Mono-trans, poly-cis decaprenyl phosphate biosynthesis	-0.669	0.253	0.012	0.265
	HOMOSER-METSYN-PWY	Biosynthesis	Amino acid biosynthesis	L-methionine biosynthesis I	-0.298	0.111	0.015	0.297
	PWY-5347	Biosynthesis	Amino acid biosynthesis	Superpathway of L-methionine biosynthesis (transulfuration)	-0.272	0.100	0.015	0.297
	METSYN-PWY	Biosynthesis	Amino acid biosynthesis	Superpathway of L-homoserine and L-methionine biosynthesis	-0.277	0.102	0.015	0.297
	GLYCOLYSIS-E-D	Generation of precursor metabolites and energy	-	Superpathway of glycolysis and the Entner-Doudoroff pathway	0.822	0.345	0.016	0.298

MET-SAM-PWY	Biosynthesis	Co-factor, carrier & vitamin biosynthesis	Superpathway of S-adenosyl-L-methionine biosynthesis	-0.260	0.098	0.018	0.300
METH-ACETATE-PWY	Generation of precursor metabolites and energy; Degradation, Utilization, Assimilation	Respiration; Acetate degradation	Methanogenesis from acetate	-0.430	0.174	0.021	0.309
PWY-8004	Generation of precursor metabolites and energy; Degradation, Utilization, Assimilation	Entner-Doudoroff pathways; Carbohydrate degradation	Entner-Doudoroff pathway I	0.762	0.336	0.021	0.309
PWY-6292	Biosynthesis	Amino acid biosynthesis	Superpathway of L-cysteine biosynthesis (mammalian)	-0.400	0.162	0.021	0.309
PWY66-399	Biosynthesis	Carbohydrate synthesis	Gluconeogenesis III	-0.416	0.173	0.024	0.328
PPGPPMET-PWY	Biosynthesis	Metabolic regulator biosynthesis	ppGpp metabolism	0.097	0.051	0.026	0.328
PWY-6471	Biosynthesis; Detoxification	Cell structure biosynthesis; Antibiotic resistance	Peptidoglycan biosynthesis IV (*Enterococcus faecium*)	-0.420	0.180	0.029	0.337
ANAEROFRUCAT-PWY	Generation of precursor metabolites and energy	Fermentation	Homolactic fermentation	-0.234	0.097	0.032	0.338
PWY-5100	Generation of precursor metabolites and energy	Fermentation	Pyruvate fermentation to acetate and lactate II	-0.175	0.070	0.033	0.338
KDO-NAGLIPASYN-PWY	Biosynthesis	Fatty acid & lipid biosynthesis; Cell structure biosynthesis	Superpathway of (Kdo)2-lipid A biosynthesis	0.612	0.298	0.035	0.340
PWY-6123	Biosynthesis	Nucleoside & nucleotide biosynthesis	Inosine-5'-phosphate biosynthesis I	0.027	0.018	0.037	0.340
CODH-PWY	Degradation, Utilization, Assimilation	C1 compound utilization and assimilation	Reductive acetyl coenzyme A pathway I (homoacetogenic bacteria)	-0.385	0.172	0.037	0.341
PWY-8187	Generation of precursor metabolites and energy; Degradation, Utilization, Assimilation	Fermentation; Amino acid degradation	L-arginine degradation XIII (reductive Stickland reaction)	-0.330	0.149	0.041	0.365
PWY-6121	Biosynthesis	Nucleoside & nucleotide biosynthesis	5-aminoimidazole ribonucleotide biosynthesis I	0.022	0.017	0.049	0.393
PWY-6124	Biosynthesis	Nucleoside & nucleotide biosynthesis	Inosine-5'-phosphate biosynthesis II	0.025	0.019	0.049	0.393

Differential abundance testing performed using MaAsLin3 v.0.99.16 linear models with total sum scaling and log transformation.
Statistical significance defined as $q \leq 0.10$ (text emboldened). Results with $p < 0.05$ and $q > 0.10$ are reported as nominal associations.
* Coefficient (Coef or β) represents the difference in the log-transformed relative abundance of each MetaCyc pathway per unit increase in continuous measures or between groups.
[#] Participants were considered to have consumed alcohol hazardously during pregnancy if AUDIT score ≥ 7.
AUDIT – Alcohol Use Disorders Identification Test; FASD – fetal alcohol spectrum disorder; HAU – hazardous alcohol use; SE - standard error; q-value – Benjamini-Hochberg false discovery rate (FDR)-adjusted *p*-value.

Table S8: Phylum-level median relative bacterial abundance of maternal vaginal swab samples in alcohol use (n = 28) and infant FASD diagnosis analysis (n = 15).

Alcohol use vaginal swab analysis (n = 28)		FASD diagnosis vaginal swab analysis (n = 15)	
**Phylum**	**Median relative abundance (%)**	**Phylum**	**Median relative abundance (%)**
Bacillota	86.90	*Bacillota*	58.20
Actinomycetota	10.30	*Actinomycetota*	15.20
Bacteroidota	1.96	*Bacteroidota*	1.23
Fusobacteriota	0.02	*Pseudomonadota*	0.001
Pseudomonadota	0.01	*Fusobacteriota*	0.001

Table S9: Median relative bacterial abundance of top five genera in maternal vaginal swab samples in alcohol use (n = 28) and infant FASD diagnosis analysis (n = 15).

Alcohol use vaginal swab analysis (n = 28)		FASD diagnosis vaginal swab analysis (n = 15)	
**Genus**	**Median relative abundance (%)**	**Genus**	**Median relative abundance (%)**
Lactobacillus	72.50	*Lactobacillus*	45.80
Gardnerella	5.28	*Gardnerella*	6.24
Prevotella	0.56	*Prevotella*	0.03
Ureaplasma	0.27	*Fannyhessea*	0.01
Hoylesella	0.21	*Ureaplasma*	0.01

The most abundant bacterial phyla present across the maternal vaginal swab samples, in order of decreasing abundance, were: *Bacillota*, *Actinomycetota*, *Bacteroidota*, *Fusobacteriota* and *Pseudomonadota* (**Table S8**).

At the genus-level, the vaginal microbiota of the 15 participants was dominated by *Lactobacillus* species, which was present at a median relative abundance of 45.8% (**Table S9**). In addition, and listed in order of decreasing median abundance, *Gardnerella* (6.2%), *Prevotella* (0.03%), *Fannyhessea* (0.01%), and *Ureaplasma* (0.01%) were among the remaining top five most abundant bacterial genera present in the samples.

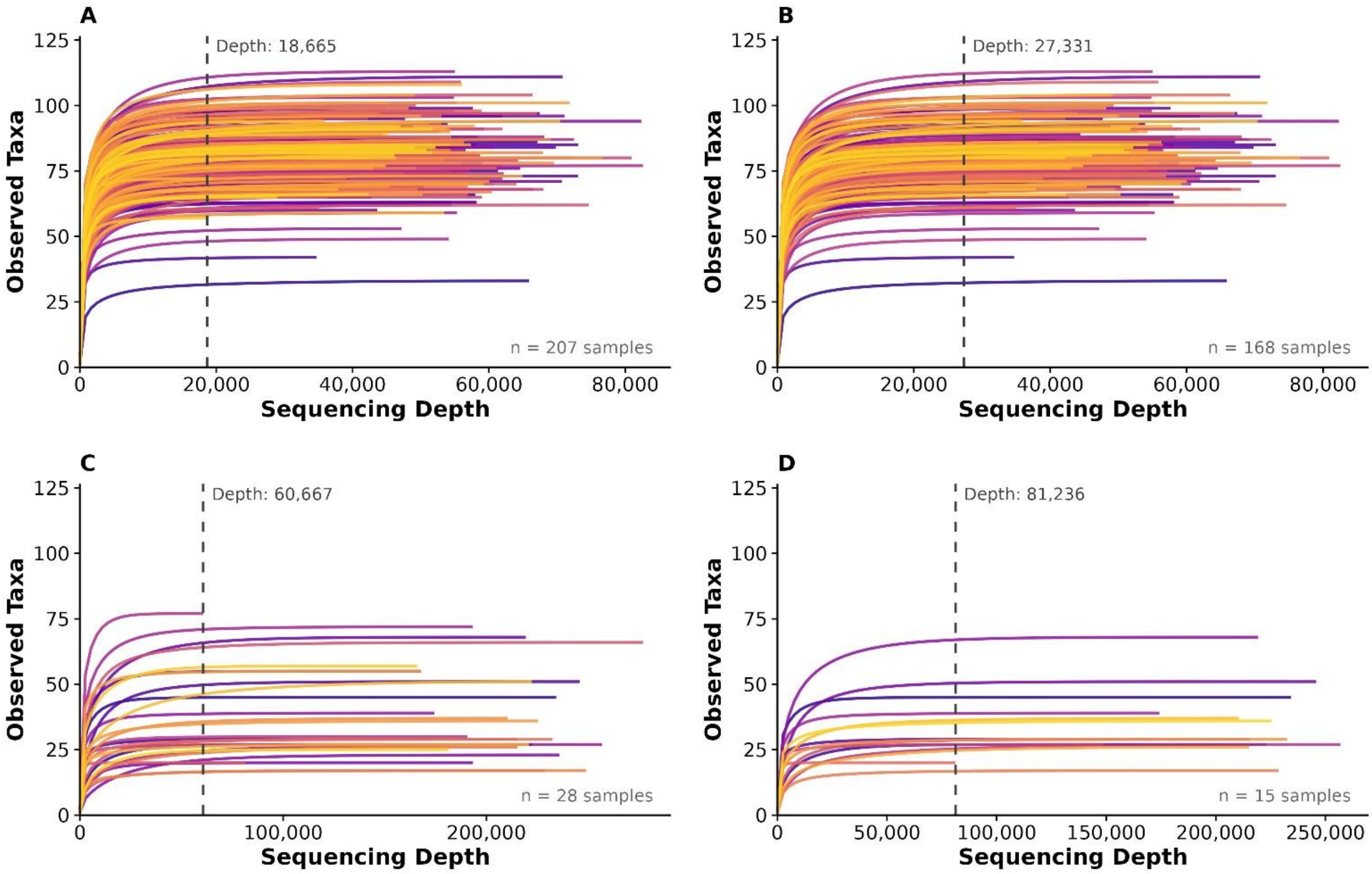

Figure S1: Sequencing depth rarefaction curves for the maternal fecal and vaginal microbiota datasets across alcohol use and infant FASD diagnosis analyses. Genus-level rarefaction curves are depicted for (**A**) fecal microbiota alcohol use analyses, (**B**) fecal microbiota infant FASD diagnosis analysis, (**C**) vaginal microbiota alcohol use analyses and (**D**) vaginal microbiota infant FASD diagnosis group analysis, conducted on fecal (n = 207) and vaginal swab (n = 28) samples collected from pregnant participants. The vertical line represents the rarefaction depth at which each dataset was sub-sampled. Reads were normalised by random sub-sampling without replacement to an even depth of 18,665 reads, 27,331 reads, 60,667 reads and 81,236 reads for the aforementioned analyses, respectively.

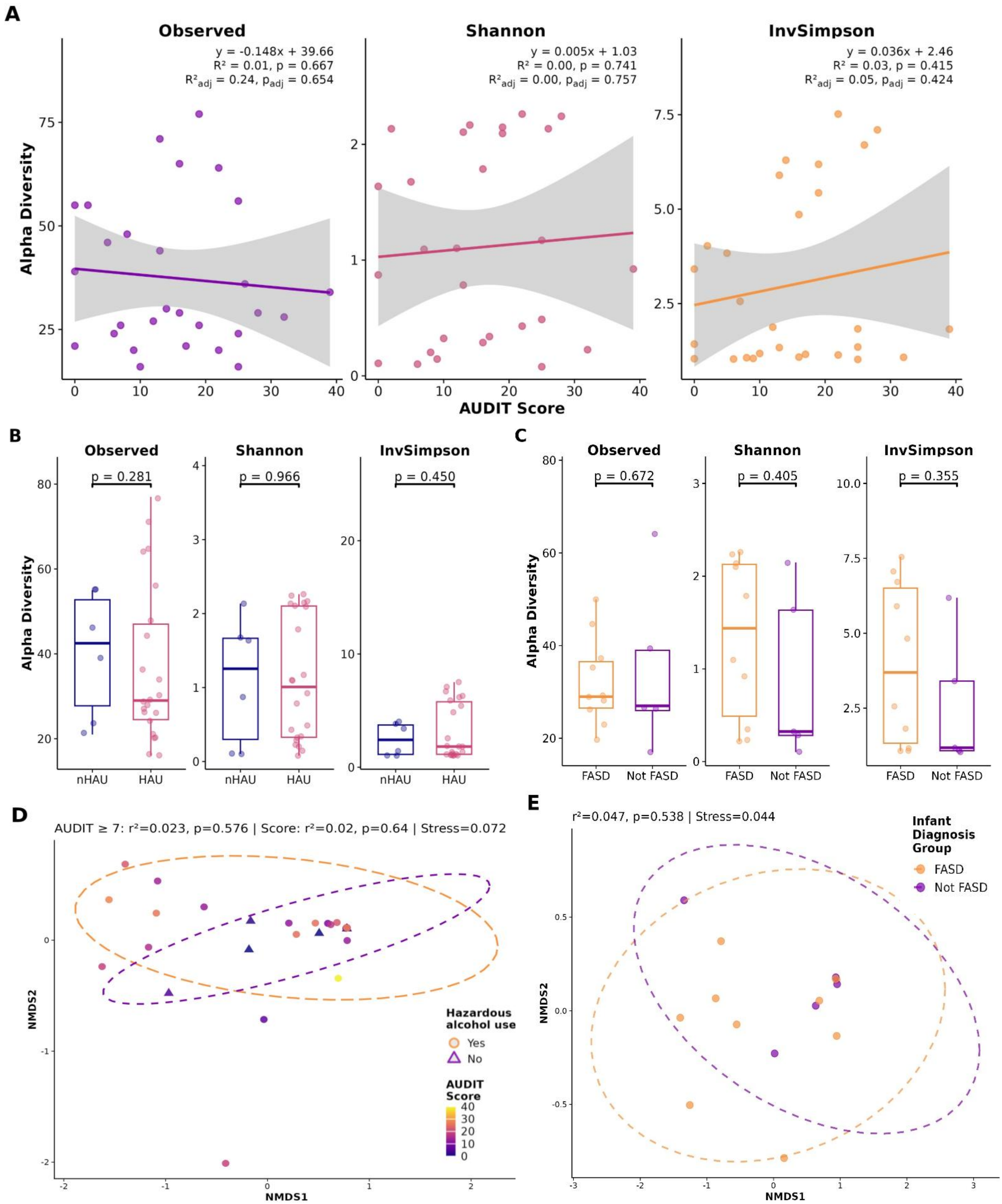

Figure S2: Maternal vaginal microbiota diversity relative to AUDIT score and infant FASD diagnosis. Linear regression and box-and-whisker plots of alpha-diversity indices (Observed richness, Shannon and Inverse Simpson) are shown in relation to (**A**) continuous AUDIT score (n = 28), (**B**) dichotomised AUDIT score reflecting HAU during pregnancy (AUDIT ≥ 7 : n = 22; AUDIT < 7: n = 6) and (**C**) infant FASD diagnostic group (FASD: n = 10; Not FASD: n = 5). NMDS Bray-Curtis dissimilarity ordination plots are shown for (**D**) investigated alcohol use measures, coloured by AUDIT score and stratified by alcohol use group, and (**E**) infant FASD diagnostic group. AUDIT – Alcohol Use Disorders Identification Test; FASD – fetal alcohol spectrum disorders; HAU – hazardous alcohol use; NMDS – non-metric multidimensional scaling.

Supporting methods: Bioinformatic analysis

Raw paired-end sequence reads were filtered using the Divisive Amplicon Denoising Algorithm 2 (*DADA2*) v.1.32.0 (Callahan et al., 2016) package using the following parameters for the gut microbiota analysis: truncLen=c(250,220), maxN=0, maxEE=c(3,3), truncQ=2, rm.phix=TRUE. The following parameters were used in the vaginal microbiota analysis: truncLen=c(240,210), maxN=0, maxEE=c(3,4), truncQ=2, rm.phix=TRUE. Filtered reads were further dereplicated, denoised, and merged using default *DADA2* parameters. Taxonomic assignment was performed using the *DADA2*-formatted SILVA SSU rRNA reference database v.138.2 (Callahan et al., 2016; Quast et al., 2013).

For the fecal microbiota analyses, a feature table comprising of 41,591 amplicon sequence variants (ASVs) derived from 207 fecal samples, with an average read length of ~ 356 bp, was constructed. For the vaginal microbiota analysis, a second independent feature table consisting of 3,846 ASVs across 28 samples, with read lengths averaging ~ 351 bp, was created. ASV tables were separately merged into a unified object with their corresponding taxonomic classifications and relevant participant metadata using *phyloseq* v.1.48.0 (McMurdie and Holmes, 2013). Sequences that remained unassigned or were not classified as “Bacteria” at kingdom-level, and those receiving “Chloroplast” or “Mitochondria” classifications at order and family level, respectively, were removed. Following filtering, 41,485 ASVs in the fecal sample dataset and 2,968 ASVs in the vaginal swab sample dataset were retained for downstream analysis. Taxa were agglomerated and investigated at the genus-level rank.

Rarefaction curves were generated using the *rarecurve* function of *vegan* (Dixon, 2003) v.2.6-10 to determine whether the minimum sequencing depth across the samples adequately captured the bacterial diversity within each dataset. The minimum read depth was found to be adequately representative of diversity, as evidenced by the plateauing of the rarefaction curves across the majority of the samples (**Figure S1**). The datasets were normalised by random sub-sampling without replacement using the *rarefy* function of the *phyloseq* (McMurdie and Holmes, 2013) package to an even depth of 18,665 and 27,331 reads for the fecal microbiota alcohol use and FASD-related analyses, and 60,667 and 81,236 reads for the vaginal microbiota alcohol use and FASD-related analyses, respectively.

Covariate adjustment sets were selected for each analysis on the basis of their independent association/s with alpha- and beta-diversity measures, statistically significant differences of variables between groups ($p \leq 0.05$) and their biological relevance. Metadata variables with too few observations for reliable statistical analysis in datasets with limited sample sizes were excluded from models. Linear regression models and permutational ANOVAs (PERMANOVAs), with 999 permutations, were used to test covariate associations with alpha-diversity metrics and beta-diversity distance matrices, and derive *p*-values and effect sizes, respectively. Covariates were retained for adjustment in downstream analyses if significantly associated with a diversity metric ($p \leq 0.05$) or if they explained ≥ 2% of variance in beta diversity ($R^2 \geq 0.02$). Covariate adjustment sets are detailed in **Table S1**.

Microbial diversity measures were generated using *vegan* (Dixon, 2003). Alpha diversity was assessed using observed richness, Shannon, and inverse Simpson diversity metrics. Differences in alpha diversity metrics between groups for the alcohol-related fecal and vaginal swab sample analysis were assessed using robust linear regression models, accounting for relevant covariate adjustment sets.

Bray-Curtis dissimilarity matrices were constructed using the metaMDS function in vegan (Dixon, 2003) and non-metric multi-dimensional scaling (NMDS) plots were generated to visualize the microbial community structure. PERMANOVAs (9,999 permutations) were performed to determine whether variables of interest were associated with differences in microbial community composition.

MaAsLin3 (v.0.99.16) (Nickols et al., 2026) was used to test associations between metadata variables and microbial community features. Analyses were run on non-rarefied data using default package parameters, with a minimum prevalence threshold of 10% and a minimum relative abundance of 0.001. In addition to the relevant covariate set for each analysis, extraction batch was modelled as a random effect and read depth was included as a covariate to account for differential sequencing depth across samples. All analyses used a two-sided test of hypothesis, a significance level of $p \leq 0.05$, and an adjusted *p*-value following Benjamini-Hochberg correction for multiple testing termed *q*. A modest significance threshold $q \leq 0.10$, chosen to enable a broader view of associations as has been performed in similar studies (White et al., 2009), was used. Genera with a nominal *p*-joint ≤ 0.05 were reported on in the absence of FDR-significant results.

PICRUSt2 (v.2.6.1) (Douglas et al., 2020) was applied to the ASV sequences of the non-rarefied gut microbiota dataset to predict gene family abundances and the related functional pathways of the

bacterial communities present. KEGG ortholog (KO) abundances were converted to KEGG pathway abundances using *ggpicrust2* (Yang et al., 2023) v.2.5.10, and MetaCyc pathway abundances were directly obtained from the *PICRUSt2* pathway inference module. Functional analysis was only performed for the fecal microbiota analysis, as functional inference tools have demonstrated poor performance when applied to vaginal samples (Carter et al., 2023). Differential abundance testing of the KEGG and MetaCyc pathways was performed using the default abundance model of *MaAsLin3*, with a minimum prevalence threshold of 10% and a minimum relative abundance of 0.001. The p-values were adjusted using Benjamini-Hochberg correction procedures, with $q < 0.10$ considered statistically significant and $p < 0.05$ reported as nominally significant for the purposes of this exploratory study.
